# Reaction of different cell types of the brain on neurotoxin cuprizone and hormone melatonin treatment in young and aging mice

**DOI:** 10.3389/fncel.2023.1131130

**Published:** 2023-04-20

**Authors:** Irina Labunets, Anzhela Rodnichenko, Sergey Savosko, Tetyana Pivneva

**Affiliations:** ^1^Cell and Tissue Technologies Department, Institute of Genetic and Regenerative Medicine, National Scientific Center “M.D. Strazhesko Institute of Cardiology”, Clinical and Regenerative Medicine of the National Academy of Medical Sciences of Ukraine, Kyiv, Ukraine; ^2^Laboratory of Pathophysiology and Immunology, D. F. Chebotarev Institute of Gerontology of the National Academy of Medical Sciences of Ukraine, Kyiv, Ukraine; ^3^Department of Sensory Signaling, Bogomoletz Institute of Physiology, National Academy of Sciences of Ukraine, Kyiv, Ukraine; ^4^Department of Biomedicine and Neuroscience, Kyiv Academic University, Kyiv, Ukraine

**Keywords:** cuprizone, melatonin, age, brain neurons and neuroinflammatory cells, multiple sclerosis, oxidative stress, bone marrow, thymus

## Abstract

**Introduction:**

The brain myelin and neurons destruction in multiple sclerosis may be associated with the production of neuroinflammatory cells (macrophages, astrocytes, T-lymphocytes) of pro-inflammatory cytokines and free radicals. The age-associated changes of the above cells can influence on the response of nervous system cells to toxic damaging and regulatory factors of humoral/endocrine nature, in particular pineal hormone melatonin. The study aim was (1) to evaluate changes of the brain macrophages, astrocytes, T-cells, neural stem cells, neurons, and central nervous system (CNS) functioning in the neurotoxin cuprizone-treated mice of different age; and (2) to assess in such mice the effects of exogenous melatonin and possible courses of its action.

**Methods:**

A toxic demyelination and neurodegeneration model was induced in 129/Sv mice aged 3–5 and 13–15 months by adding cuprizone neurotoxin to their food for 3 weeks. From the 8th day of the cuprizone treatment, melatonin was injected intraperitoneally at 6 p.m. daily, at a dose of 1 mg/kg. The brain GFPA + -cells were evaluated by immunohistochemical method, the proportion of CD11b+, CD3+CD11b+, CD3+, CD3+CD4+, CD3+CD8+, Nestin+-cells was determined via flow cytometry. Macrophage activity was evaluated by their ability to phagocytose latex beads Morphometric analysis of the brain neurons and the behavioral reactions (“open field” and rotarod tests) were performed. To assess the involvement of the bone marrow and thymus in the action of melatonin, the amount of granulocyte/macrophage colony-forming cells (GM-CFC), and blood monocytes and thymic hormone thymulin were evaluated.

**Results and discussion:**

The numbers of the GFAP+-, CD3+-, CD3+CD4+, CD3+CD8+, CD11b+, CD3+CD11b+, Nestin+-cells and macrophages phagocytic latex beads and malondialdehyde (MDA) content were increased in the brain of young and aging mice under cuprizone influence. The proportion of undamaged neurons within the brain, motor, affective, and exploratory activities, and muscle tone decreased in mice of both ages. Introducing melatonin to mice of any age reduced the number of GFAP+-, CD3+- cells and their subpopulations, macrophage activation, and MDA content. At the same time, the percentage of brain neurons that were unchanged increased as the number of Nestin+ cells decreased. The behavioral responses were also improved. Besides, the number of bone marrow GM-CFC and the blood level of monocytes and thymulin increased. The effects of both neurotoxin and melatonin on the brain astrocytes, macrophages T-cells, and immune system organs as well as the structure and functioning of neurons were more pronounced in the young mice.

**Conclusion:**

We have observed the involvement of the astrocytes, macrophages, T-cells, neural stem cells, and neurons in the brain reaction of mice different age after administration of neurotoxin cuprizone and melatonin. The brain cell composition reaction has the age features. The neuroprotective effects of melatonin in cuprizone-treated mice have been realized through an improvement of the brain cell composition and oxidative stress factors and functioning of bone marrow and thymus.

## Introduction

Multiple sclerosis is one of the most common demyelinating pathologies of the central nervous system (CNS) (Mishchenko et al., 2014; Vaughn et al., 2019). This results in the loss of myelin in nerve fibers, disruption of nerve impulse transmission, and a decline in motor activity. Recently, the leading scientists consider multiple sclerosis as a neuro-degenerative pathology. Destruction of neurons in different areas of the CNS can lead to the changes of their functioning and, as a result, to the disorders not only in motor activity but also in emotions, memory, vegetative dysfunction, intellect, etc.

Multiple sclerosis usually affects younger people, but today it can be reported in people over the age of 45. In older patients, this disease has pre-dominantly infectious and toxic origin, progressive character and more severe clinical course (Vaughn et al., 2019).

The pathogenic links of neuronal and myelin destructions in multiple sclerosis can be the productions of oxidative stress and neuro-inflammation (Chen et al., 2020; Absinta et al., 2021; Murioz-Jurado et al., 2022). For example, the brain microglial cells and macrophages produce free radicals and pro-inflammatory cytokines (TNF-α, IFN-γ, and IL-1β) (Li and Barres, 2018). During neuro-inflammation the T-lymphocytes infiltrate the brain and become source of pro-inflammatory cytokines (Sonobe et al., 2007; Gonzalez and Pacheco, 2014).

In addition to microglia inflamed in multiple sclerosis, large populations of reactive astrocytes and stressed oligodendrocytes were present on the chronic active lesion area. As is known, astrocytes act a crucial role in the pathogenesis of multiple sclerosis. They take part in regulating the blood–brain barrier (BBB); in modulating T cell activity through the production of cytokines; in expressing toll-like receptors and major histocompatibility complex class I and II (Burda and Sofroniew, 2014; Liddelow and Barres, 2015; Sen et al., 2022).

Age-associated changes both in the amount of macrophages, microglial cells, T-cells, astrocytes and their relationships can influence not only on the efficiency of restoration of neurogenesis after its injury but also on the response of nervous system cells to regulatory factors of cellular/humoral nature (Gemechu and Bentivoglio, 2012; Hamilton et al., 2013; Moraga et al., 2015; Labunets et al., 2017a). Investigation of age aspects of nervous cells reaction on the neurotoxic and regulatory factors is impossible without the use of adequate experimental model of multiple sclerosis.

One of them is cuprizone toxic model. Neurotoxin cuprizone is a copper chelator that decreases cytochrome and monoamine oxidase activities in the mitochondria of mature oligodendrocytes and, as a result, leads to apoptosis in these cells and demyelination in CNS (Praet et al., 2014; Vega-Riquer et al., 2019; Zhan et al., 2020). According to our previous data, the cuprizone induced degeneration the neurons in cerebral cortex and cerebellum of experimental mice (Labunets et al., 2017a, 2018a,b; Labunets and Rodnichenko, 2020). According to the literature, neuroinflammation, and oxidative stress are important for oligodendrocyte and neuron damage in cuprizone-treated young animals (Gudi et al., 2014; Praet et al., 2014; Vega-Riquer et al., 2019).

Among the endocrine regulatory factors, the pineal gland hormone melatonin shows a wide range of biologic activity in the organism. This hormone acts on immune (thymus, bone marrow, macrophages, lymphocytes, etc.) and endocrine (pituitary, adrenal, gonadal glands, etc.) systems functions, regulates the biological rhythms in organism and reveals antiapoptotic, antioxidant, anti-inflammatory, and neurotrophic effects (Sarlak et al., 2013; Csaba, 2016; Wurtman, 2017; Murioz-Jurado et al., 2022). Melatonin synthesis undergoes changes with age and in experimental demyelination (Hardeland, 2012; Wurtman, 2017). In contrast, the exogenous melatonin exhibits neuroprotective effects in animals of different age with experimental models of nervous system pathology (Hardeland, 2012; Murioz-Jurado et al., 2022). The aim of study was:(1) to evaluate changes of the brain macrophages, astrocytes, T-cells, neural stem cells, neurons and CNS functioning in the neurotoxin cuprizone-treated mice of different age; and (2) to assess in such mice the effects of exogenous melatonin and possible courses of its action.

## Materials and methods

### Animals

Experiments were performed on 3–5-month-old (young, *n* = 79) and 13–15-month-old (aging, *n* = 79) female 129/Sv mice (H-2b genotype) from experimental clinic of the Institute of Genetic and Regenerative Medicine. According to our investigations, the 129/Sv mice of both sexes are sensitive to the toxic action of neurotoxin cuprizone as well as C57Bl/6 mice where widely used in such experiments (Labunets et al., 2014a,b; Labunets, 2018). The animals were kept in standard vivarium conditions with a fixed light regimen 12:12 with food and water *ad libitum*. Biological tissues for studies were taken at 9 a.m. by ether anesthesia.

All experiments were carried out according to the European Convention for the Protection of Vertebrate Animals used for Experimental and other Scientific Purposes [European Union Directive of 22 September 2010 (2010/63/EU)] and Article 26 of the Law of Ukraine “On the Protection of Animals from Cruelty” (No.3447-IV, 2006) and were approved by the Bioethics Committee of Institute of Genetic and Regenerative Medicine.

### Experimental groups of mice

Experimental animals were grouped (i) intact young (*n* = 21) and aging (*n* = 21) animals, receiving standard food; (ii) young (*n* = 21) and aging (*n* = 21) mice, receiving cuprizone-containing food for 3 weeks and injections of solvent; (iii) young (*n* = 21) and aging (*n* = 21) mice with cuprizone diet and melatonin injection; (iv) young mice (*n* = 16) and aging (*n* = 16) mice with standart food (intact mice) which received both melatonin and solvent. Experiments were conducted after completion of cuprizone diet on 7, 21, and 60 days.

### Cuprizone-induced model of demyelination and neurodegeneration

The mice of both age groups received the neurotoxin cuprizone [bis(cyclohexylidenehydrazide)] (Sigma-Aldrich, USA). The animals were fed food mixed with cuprizone at 0.2% (w/w), daily for 3 weeks (Praet et al., 2014; Labunets et al., 2018a,b; Labunets and Rodnichenko, 2020; Zhan et al., 2020). Intact animals used regular food. The body weight of each mice was measured before and after cuprizone administration alone or in its combination with melatonin.

Weight loss is one of the signs of the toxic effects of cuprizone. After cuprizone treatment the young and aging mice lost weight. Before cuprizone treatment, body weights of young and aging mice were 22.0 ± 1.4 and 28.1 ± 1.9 g, respectively. After completion of the cuprizone diet, 18.0 ± 1.1 and 23.1 ± 1.2 g, respectively (*p* < 0.05). Weight of mice with cuprizone and melatonin treatment did not changed (data not shown).

### Melatonin injections

Cuprizone-treated mice of both age groups received melatonin i.p. injections (Sigma-Aldrich, USA) in the dose of 1 mg/kg every day at 6 p.m. starting from the 8th to 21st day of the cuprizone diet. Melatonin was dissolved in solvent (0.9% sodium chloride). After 8–10 days of cuprizone diet the signs of both apoptosis of oligodendrocytes and structural changes of the brain neurons developed in mice of different strains, including 129/Sv (Praet et al., 2014; Labunets et al., 2018a; Zirngibl et al., 2022). In addition, we also evaluated the effects of melatonin alone on some parameters (thymus and bone marrow functions) in mice of both ages group (*n* = 8) with regular diet. Melatonin was administered according to the above scheme. Control groups of mice received solvent (sodium chloride) injections (*n* = 8).

### Immunophenotyping of the brain cells

Immunophenotyping for CD3+, CD4+, CD8+ and CD11b+ (Mac-1) markers was performed using the mouse monoclonal antibodies conjugated to fluorochromes (BD Biosciences, USA): CD3 – PE-conjugated antibodies (cat. no. 555275), CD4 – APC-Cy™ 7 conjugated antibodies (cat. no. 552051), CD8-APC conjugated antibodies (cat. no. 553035) and CD11b – FITC-conjugated antibodies (cat. no. 557396). The number of brain neural stem cells was determined by the expression of Nestin (Lendahl et al., 1990). To identify Nestin + cells, we used PE-conjugated mouse anti-nestin monoclonal antibodies (BD Biosciences, USA, cat. no. 561230). All antibodies used at a concentration of 0.5 μg/ml. The whole brain was homogenized; passed through the filter (70 μm); cells were fixed for 10 min at room temperature with 4% solution of paraformaldehyde in 0.1 M phosphate buffered saline (pH = 7.4); permeabilized and stained in Perm/Wash buffer (Becton Dickinson, USA) according to the manufacturer’s instructions. Cell samples without antibodies were used as a control. The measurements of the percentage of labeled cells were performed using a flow cytometer-sorter BD FACSAria™ I and BD FACS Diva 6.1 software (Becton Dickinson, Franklin Lakes, NJ, USA).

### Functional activity of brain macrophages/phagocytic cells

Brain macrophages are heterogeneous population (Jordan and Thomas, 1988; Jordan et al., 1990). Among these cells, microglia, and blood macrophages have a similar property, namely, the ability to phagocytose latex beads. The activity of macrophage/phagocytic brain cells in our experiments was determined according to Jordan et al. (1990) and in our modification (Labunets et al., 2017a). The method is based on the quantitative determination of the latex beads that are phagocytosed by activated macrophages after their incubation.

Whole-brain cell suspensions (*n* = 5 from each groups of mice) were first passed through a series of 100 μm and then 70 μm cell filters (Sigma-Aldrich, USA). Then cells were transferred to Petri dishes (100 mm diameter) in culture medium consisting of RPMI-1640, 10% fetal bovine serum, 2 mM L-glutamine, penicillin 100 U/ml, streptomycin 100 μg/ml (all reagents Sigma-Aldrich, USA). After cultivation for 1 h in CO_2_ incubator in humidified atmosphere with 5% CO_2_ at a temperature of 37°C the adherent cells were dissociated with 0.05% trypsin in 0.53 mM Na_2_EDTA (Sigma-Aldrich, USA). Then 0.2 ml of cells suspension (2.5 × 10^6^/ml) was placed on microscope slides and incubated for 1 h in the CO_2_ incubator. After the cells incubation 0.2 ml suspension latex (2.5 × 10^8^/ml) (Sigma-Aldrich, USA) was added and then incubated for 45 min. Then the cells were fixed and stained with Romanovsky-Gimsa (Macrochim, Ukraine). A total of 100 macrophages/phagocytes were counted and measured under a light microscope: (a) percentage of brain macrophages capable of phagocytosis of latex beads (phagocyte index) and (b) number of latex beads that phagocytose one macrophage (phagocyte number).

### Immunohistochemistry

After decapitation, the removed brains (*n* = 5 from each groups of mice) were fixed in phosphate-buffered (PB) paraformaldehyde 4% in 0.1 M PB, pH 7.4. The sagittal sections of the brain (50 μm thick) were cut on a vibratome VT1000A (Leica, Wetzlar, Germany). Immunohistochemical staining of astrocytic cells was performed as previously described (Zabenko and Pivneva, 2016). Then hippocampus slices were washed with 0.1 M PB and blocked in 0.1 M PB containing 0.3% Triton X-100 and 0.5% bovine serum albumin. To identify the astrocytic cells the following primary antibody was used: polyclonal rabbit anti glial fibrillary acidic protein [GFAP, 1:1,500; Z0334^1^ (GFAP) Dako cat z0334/product/Agilent technologies, DakoCytomation, Glostrup, Denmark]. On the next day sections were incubated with the secondary anti-rabbit antibody Alexa Fluor 594 (1:1,000, A-11012, Molecular Probes Inc., USA) diluted in 0.1 M PB, 0.5% bovine serum albumin, 0.3% Triton X-100. The slices were mounted on glass slides with fluorescent mounting medium Immu-Mount (Thermo Scientific, Waltham, MA, USA). Confocal images of glial cells were acquired with a laser-scanning microscope (FV1000-BX61WI, Olympus, Tokyo, Japan). For the evaluation of cells, the program Image J (NIH, USA) was used. The number of GFAP+ cells per 0.1 mm^2^ area was counted in the CA1zone of the stratum radiatum of the hippocampus.

### Morphological studies of cerebral cortex neurons

Brain samples for morphological studies were obtained after transcardial perfusion of mice with 4% paraformaldehyde solution in 0.1 M PB (pH = 7.4). After dehydration in ascending alcohol solutions, brain samples were embedded in paraffin and sliced at 8 μm thickness in the frontal position of the brain. Paraffin sections were deparaffinized and rehydrated; were stained with toluidine blue for 10 min, were washed with tap water, differentiated with 1% glacial acetic acid, and washed again with tap water (Lu et al., 2022). After washing, the slices were cleared with ethanol and xylene for a few minutes, and mounted with Histofluid Mounting Media (Paul Marienfeld GmbH & Co. KG). Toluidine blue selectively stains acidic tissue components such as DNA in nuclei and RNA cytoplasm (Nissl bodies) of neuron and helps to analysis cell death and function (protein synthesis or functional degradation). Microphotographs of neurons were taking using a microscope Olympus BX-51.

Neurons were measured in layers III-V of cortex. The neurons were grouped into three categories: first – damaged neurons. Criteria for neuronal damage were the following: cytolysis, nucleolysis and nuclear pycnos, hydropic dystrophy, and/or deformed perikaryon. These neurons were considered as cuprizone-induced damage (destructive changes); second – neurons with moderate changes. These neurons were characterized by increased nucleus size, and the cytoplasm of the neurons was hypochrome (Labunets et al., 2017a, 2018a,2018b). Third group of neurons were with no signs of damage (no changes). The percentage of all neurons in the total number of neurons in layers III–V of the cortex was calculated from 10 histologically prepared images of mice from each experimental group, both young (*n* = 5) and aging (*n* = 5).

### Malondialdehyde

The content of malondialdehyde (MDA) in whole brain homogenate was determined by the color intensity of the trimethine complex formed between thiobarbituric acid and MDA (Uchiyama and Mihara, 1978). The content of MDA was determined spectrophotometrically (μQuant Spectrophotometer, BioTek, USA) for *n* = 5 mice in each group.

### Granulocyte/macrophage colony-forming cells

Granulocyte/macrophage colony-forming cells (GM-CFCs) were evaluated in the semi-agar cultures (Bradley and Metcalf, 1966; Labunets et al., 2017b). Bone-marrow cell suspensions were prepared by flushing from femur with R+PMI-1640 medium according to Anjos-Afonso and Bonnet (2008). 3 × 10^5^ bone marrow cells in 0, 1 ml in RPMI-1640 were added to 1 ml medium McCoy 5A supplemented with 15% fetal bovine serum, 10 mM of L-glutamine, 1.6% sodium pyruvate, 0.94% of sodium bicarbonate, 20 mM of HEPES, 1% granulocyte/macrophage-colony-stimulating factor (at a final concentration 0.5 ng/ml) (all reagents – Sigma-Aldrich, USA). Cultivation of bone-marrow cells was performed for 9 days in CO_2_ incubator in humidified atmosphere with 5% CO_2_ at a temperature of 37°C. On the 9th day of cultivation the number of GM-CFCs colonies consisting of at least 50 cells was counted. The types of colonies were following: granulocytic, mixed, and macrophage (Labunets et al., 2017b). The results were expressed as relative (per 10^6^ bone marrow cells) and absolute number of GM-CFCs in the bone marrow of one femur in each experimental groups of mice (*n* = 5).

### Peripheral blood monocytes

Calculation of the leukocyte formula of the peripheral blood of animals was carried out by the generally accepted method (Stephanov, 2001). This method is based on morphological recognition and counting of different cell types (neutrophils, eosinophils, basophils, lymphocytes, and monocytes) in stained peripheral blood smears. The smears were stained with Romanovsky-Gimsa stain (Macrochim, Ukraine). In the stained smears we found 200 leukocytes and individual cell types were expressed as a percentage.

### The blood level of thymulin/thymic serum factor

Serum samples obtained by centrifugation of clotted blood were stored at −20°C. Thawed samples were filtered through Centriflo CF-50A ultrafilter (Amicon, USA) to remove the high molecular inhibitor of thymulin. The determination method of level thymulin is based on the its ability to restore the sensitivity of spontaneous rosette formation by splenocytes from thymectomized mice to inhibition with azathioprine (Sigma-Aldrich, USA), as described (Bach et al., 1978; Labunets et al., 2017b). Thymulin levels were evaluated at the last serum dilution, which reduced the number of rosette-forming cells by 50% compared to controls (cells without serum). The results were recorded as log_2_ thymulin titer.

### Behavioral phenomena in animals

Behavioral reactions in mice were studied using the “open field” and rotarod tests. These tests provide adequate estimate of behavioral phenomena in young and aging animals (Franco-Pons et al., 2007; Gorina et al., 2017). The “open field” test allows to evaluate the locomotor activity (the number of crossed squares), the emotional activity (the number of fecal boluses) and the exploratory activity (the number of rearing and hole peeking). As shown, behavioral responses in mice are similar to human behavioral responses in neuropsychiatric disorders (Fisch, 2007). The duration of the “open field” test in mice (*n* = 15) in each groups was 3 min.

The rotarod test can assess an animal’s coordination of movement, balance, and muscle tone. During the test (10 min), the rotation speed was changed sequentially from 10 rpm (3.0 V, 300 mA) to 20 rpm (5.0 V, 300 mA). Data were presented as total residence time (s) of mice (*n* = 10 in each groups) on the rod.

### Statistical analysis

All data are presented as mean ± SEM. Statistical analysis was performed using StatSoft Statistica software 7.0 (StatSoft Inc., USA). Students two tailed *t*-test (paired or unpaired) was used to determine statistical differences between different experimental groups where appropriate. A *p* < 0.05 was considered as statistically significant.

## Results

### Changes in various cell types in brains of young and aging mice after treatment with cuprizone and melatonin

#### Brain T cells

One of early changes in the composition of the brain cells in cuprizone-treated mice (5–7 days of neurotoxin use) is the apoptosis of oligodendrocytes and structural changes in the neurons (Gudi et al., 2014; Labunets et al., 2018a).

**FIGURE 1:**
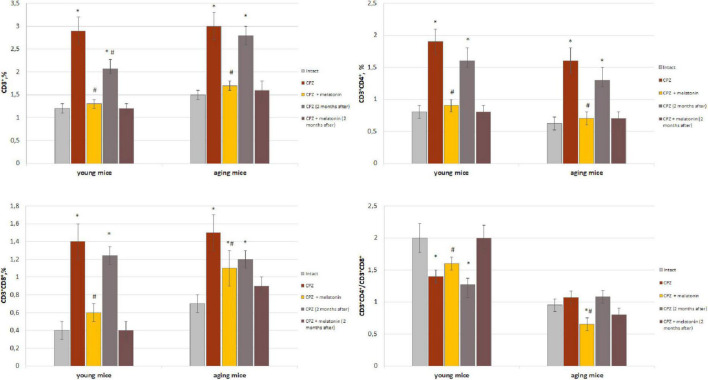
Percentage of CD3+, CD3+CD4+, and CD3+CD8+ T cells from total brain cells and cell ratio of CD3+CD4+/CD3+CD4+ in brain of young and aging mice of experimental groups (*n* = 5 in each group). Represented data are in 3 weeks of cuprizone (CPZ) diet and in 2 months after. Data are *M* ± SEM. *p < 0.05 compared to intact group; ^#^*p* < 0.05 compared to CPZ (*t*-test).

We found that the amount of CD3+ T cells in the brain of mice of different group of age after 7 days of cuprizone diet (in each group *n* = 4) did not differ from that in intact animals (data not shown). As shown in Figure 1, the amounts of CD3+, CD3+CD4+ and CD3+CD8+ cells in the brain of young and aging cuprizone-treated mice (within 3 weeks) were significantly higher than in intact mice of the same age. Melatonin injection reduced the number of cell types in both age groups and their levels reached the values observed in the intact animals (except CD3+CD8+ cells in aging mice). The ratio of CD3+CD4+/CD3+CD8+ cells in the brains of young mice treated with cuprizone was reduced compared to intact animals and increased after melatonin administration to compare with cuprizone-treated mice (Figure 1). In aging mice, the cells ratio value did not change after cuprizone treatment but decreased after melatonin administration.

We have showed that 2 months after the completion of a 3-week cuprizone diet (period of remyelination and recovery of altered functions) the amount of CD3+ T cells and their subpopulations in the brain of young mice decreased but was still higher compared to intact mice (Figure 1). Two months after completion of cuprizone and melatonin treatment the amount of CD3+, CD3+CD4+ and CD3+CD8+ T cells corresponded the values in the intact animals.

In aging mice, 2 months after the completion of cuprizone treatment the amount of CD3+, CD3+CD4+ and CD3+CD8+ cells remained higher than in intact mice (Figure 1).

Two months after the end of cuprizone and melatonin treatment, the number of above cells did not differ from those immediately after using neurotoxin and hormone (Figure 1).

Thus, the number of T cell subpopulations in the brains of young and aging mice change in response to the effect of the cuprizone. Conversely, exogenous melatonin has a positive effect on these changes. The effect of melatonin on cuprizone-induced T cells changes persist for a long time. The influence of cuprizone and melatonin on T cells changes is more pronounced in young mice.

#### Brain macrophages

**FIGURE 2:**
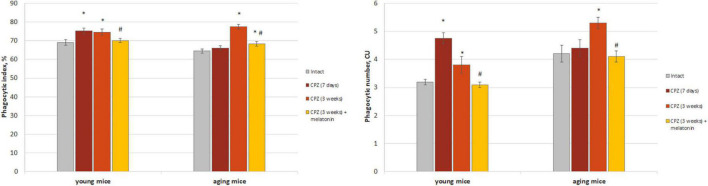
The amount (phagocytic index) and activity (phagocytic number) of macrophages phagocytic latex beads in the brain of young and aging mice of experimental groups (*n* = 5 in each group). Represented data are in 7 days and 3 weeks of cuprizone (CPZ) diet. Data are *M* ± SEM. *p < 0.05 compared to intact group; ^#^*p* < 0.05 compared to 3 weeks of CPZ (*t*-test).

We found that both the index of latex beads phagocytic macrophages and of their phagocytic number in the brain of young mice after 7 days of taking cuprizone diet was higher than in the intact animals and remained that after 3 weeks of cuprizone diet (Figure 2). In the brain of aging mice, the index of latex beads phagocytic macrophages and their phagocytic number increased only after 3 weeks of cuprizone supplementation. After melatonin injections the values of the studied parameters decreased in mice of both age groups compare to cuprizone treated mice.

**FIGURE 3:**
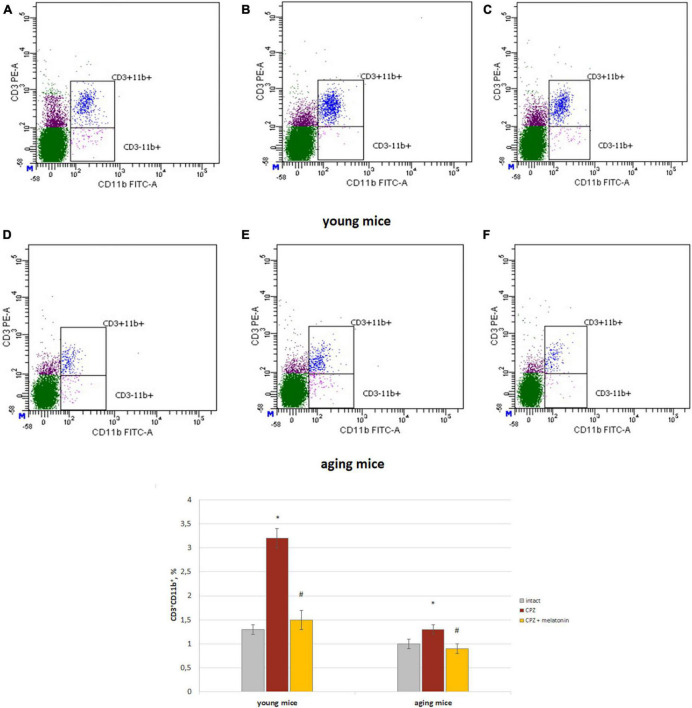
Histograms of expression of CD3 and CD11b markers in the whole brain cells according to flow cytometry and the percentages of CD3+CD11b+ cells in the brain of young and aging mice of experimental groups (*n* = 5 in each group). **(A,D)** Intact groups; **(B,E)** cuprizone treated groups; **(C,F)** cuprizone + melatonin groups. Represented data are in 3 weeks of cuprizone (CPZ) diet. Data are *M* ± SEM. *p < 0.05 compared to intact group; ^#^*p* < 0.05 compared to CPZ (*t*-test).

The amount of CD3+CD11b+ cells (activated macrophages) increased in both of young and aging neurotoxin-treated mice compare to intact groups of mice. After melatonin injections, the values of the studied indicators did not differ from those in the intact mice of both age groups (Figure 3).

Thus, we observed changes in both of the phagocytic index and phagocytic number of brain macrophages in response to cuprizone effect regardless of the age of mice. Exogenous melatonin reduced the values of the studied parameters in the cuprizone-treated mice.

#### Brain astrocytes

**FIGURE 4:**
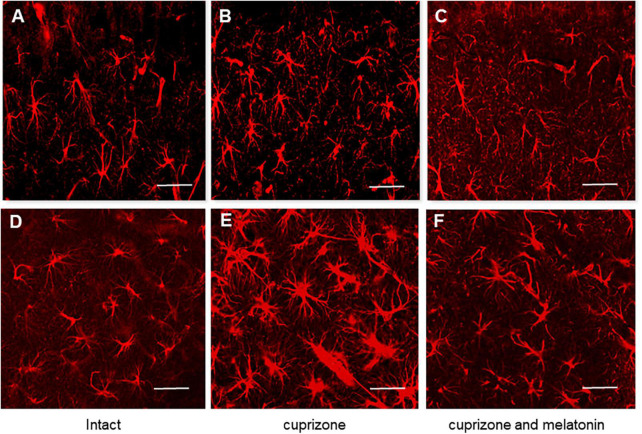
Confocal images of immunohistochemical staining of hippocampal CA1 area in intact **(A,D)**, cuprizone treated **(B,E)** and **(C,F)** cuprizone + melatonin treated mice. Immunohistochemical staining using the astrocyte marker GFAP in the hippocampal slices (*n* = 10) of mice (*n* = 5 in each group). **(A–C)** Young mice, **(D–F)** aging mice. Note a prominent outgrowth of GFAP-positive cells in panels **(B,E)** after cuprizone treatment (3 weeks). Scale bar, 50 μm.

To study the reactivity of astrocytes after cuprizone and cuprizone + melatonin treatment, we carried out immunohistochemical labeling for the marker of astrocytes (GFAP). Our immunohistochemical studies have shown that the number of GFAP-positive astrocytes in the hippocampal CA1area was in both intact young and aging animals (21.40 ± 4.19, 21.50 ± 4.09), respectively. GFAP-positive astrocytes had non-enlarged soma with thin processes by their morphology (Figures 4A, D). After cuprizone use, the numbers of GFAP-positive astrocytes were markedly increased in all layers of the CA1 area in young and aging mice (37.63 ± 9.02 and 46.30 ± 6.67, respectively). The astrocytes acquired a hypertrophied form of both the soma and the processes (Figures 4B, E). It is known that the main manifestations of reactive gliosis are hypertrophy of astrocyte processes and upregulation of GFAP in their processes (Absinta et al., 2021).

After melatonin treatment the number of GFAP-positive astrocytes significantly decreased compared with those in cuprizone treatment and achieved 26.00 ± 5.81 in young and 28.70 ± 5.29 in aging animals but did not reach the control values (Figures 4C, F).

Thus, hormone melatonin significantly reduced astrocyte activation in the young and aging mice due to the antiapoptotic, antioxidant, anti-inflammatory, and neurotrophic effects (Wurtman, 2017).

#### Brain Nestin+ cells and neurons

**FIGURE 5:**
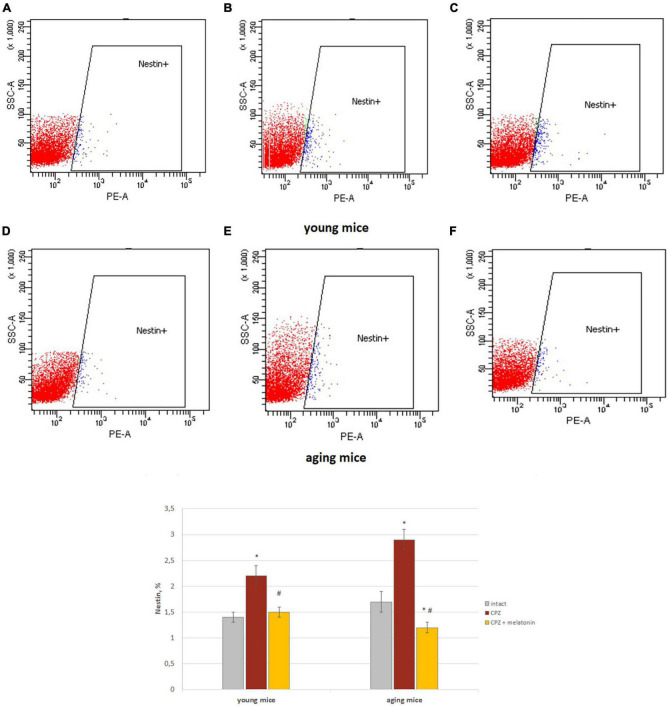
Histograms of Nestin marker expression in the total brain cells according to low cytometry and the percentage of Nestin+ cells in the brain of young and aging mice of each experimental groups (*n* = 5). **(A,D)** Intact groups; **(B,E)** cuprizone groups; **(C,F)** cuprizone + melatonin groups. Represented data are in 3 weeks of cuprizone (CPZ) diet. Data are *M* ± SEM. *p < 0.05 compared to intact group; ^#^*p* < 0.05 compared to CPZ (*t*-test).

We showed that the number of Nestin+ cells in the brains of young and aging mice treated with cuprizone was higher than in intact mice (Figure 5). After injections of melatonin, the number of Nestin+ cells decreased in mice of both ages compared to cuprizone-treated mice (Figure 5).

**FIGURE 6:**
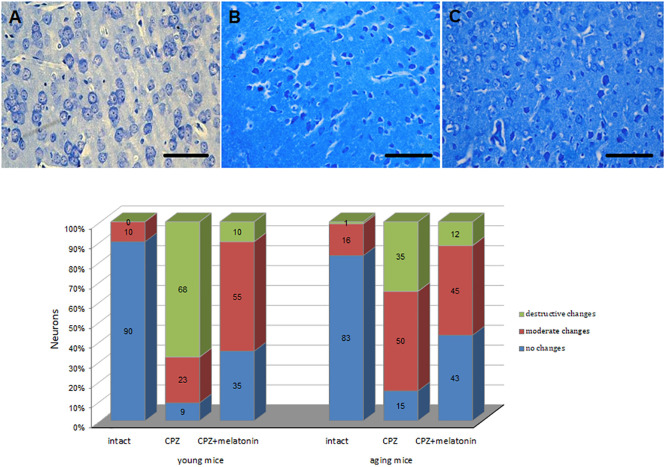
Microphotographs of histological sections of cerebral cortex (*n* = 10) and the percentages of different types of neurons in cerebral cortex of young and aging mice of experimental groups (*n* = 5 in each group): **(A)** intact group; **(B)** cuprizone group; **(C)** cuprizone + melatonin group (Labunets et al., 2018b). Toluidine blue staining. Scale bar, 50 μm.

The percentage of unchanged neurons in cerebral cortex of cuprizone-treated mice of both ages decreased, while the percentage of neurons with destructive and moderate structural changes increased compared to the intact group (Figure 6). The percentage of neurons with destructive changes was higher in young cuprizone-treated mice than in aging mice. Injections of melatonin in both young and aging mice increased the percentage of unchanged neurons and decreased the percentage of neurons with destructive changes.

Thus, in the brains of young and aging mice, cuprizone changes the number and activity of neuroinflammatory cells (macrophages, astrocytes, and T cells) on the one hand and the structure of neurons on the other hand. This cellular imbalance was more pronounced in young cuprizone-treated animals. Exogenous melatonin has positive effects on T cells, macrophages, astrocytes and structurally altered neurons in the brains of cuprizone-treated mice of different ages. Effect was more pronounced in young mice.

### Changes of behavioral reactions in young and aging mice after treatment with cuprizone and melatonin

An important manifestation of structural changes in neurons in the pathology of the nervous system was their dysfunction (Franco-Pons et al., 2007; Dutta and Trapp, 2011; Labunets et al., 2017a).

**FIGURE 7:**
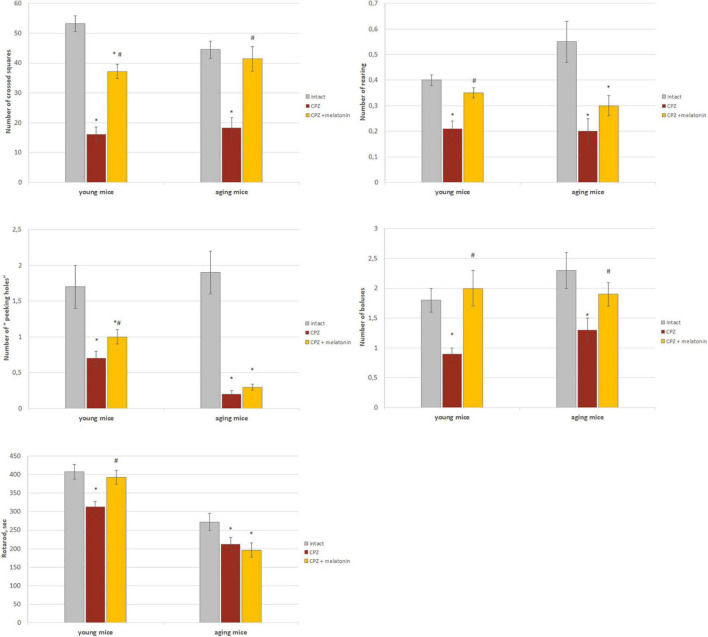
The effect of cuprizone (CPZ) and CPZ + melatonin on behavioral indicators (“open field” and rotarod tests) in young and aging mice of experimental groups (*n* = 15 in each group). Represented data are in 3 weeks of CPZ diet. Data are *M* ± SEM. *p < 0.05 compared to intact group; ^#^*p* < 0.05 compared to CPZ (*t*-test).

As shown in Figure 7, behavioral indicators in the “open field” test of young and aging mice treated with cuprizone were significantly lower than that of intact mice. The number of crossed squares increased after the injections of melatonin in mice of both ages. Moreover, values in aging mice reached values found in intact animals. Peeking and rearing activity increased after melatonin administration only in young cuprizone-treated mice. The number of boluses increased in mice of both age groups after administration of melatonin and did not differ from intact mice.

Rotarod test indicators were lower in young and aging mice treated with cuprizone compared to intact animals (Figure 7). After administration of melatonin, this behavioral parameter reached intact mice values only in young cuprizone-treated animals.

Thus, treatment with cuprizone reduces behavioral reactions in young and aging mice. Melatonin injections improve all behavioral parameters in young mice but only motor and emotional activity in aging mice.

### Effects of cuprizone and melatonin treatment on brain oxidative stress and bone marrow and thymus function

#### Brain oxidative stress

One of the factors of oxidative stress is content of MDA. It is formed as a result of peroxidation of polyunsaturated fatty acids and can react with nucleic acids, phospholipids and amino acids.

**FIGURE 8:**
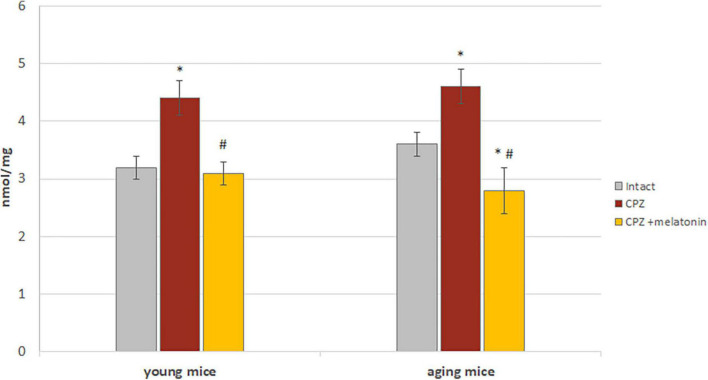
The effects of cuprizone (CPZ) and CPZ + melatonin on malondialdehyde content in the brain of young and aging mice of experimental groups (*n* = 5 in each group). Represented data are in 3 weeks of CPZ diet. Data are *M* ± SEM. *p < 0.05 compared to intact group; ^#^*p* < 0.05 compared to CPZ (*t*-test).

Higher content of MDA was found in the brains of young and aging mice on the cuprizone diet compared to intact animals (Figure 8). After administration of melatonin, MDA content decreased to that of intact mice (Figure 8).

Thus, the results confirmed the antioxidant effect of melatonin on the brains of young and aging mice treated with cuprizone.

#### GM-CFCs in bone marrow and monocytes in the blood

It has been shown that the number of macrophages in the brain can be increased due to circulating monocytes derived from progenitor cells- bone marrow GM-CFCs (Zhao et al., 2018).

**FIGURE 9:**
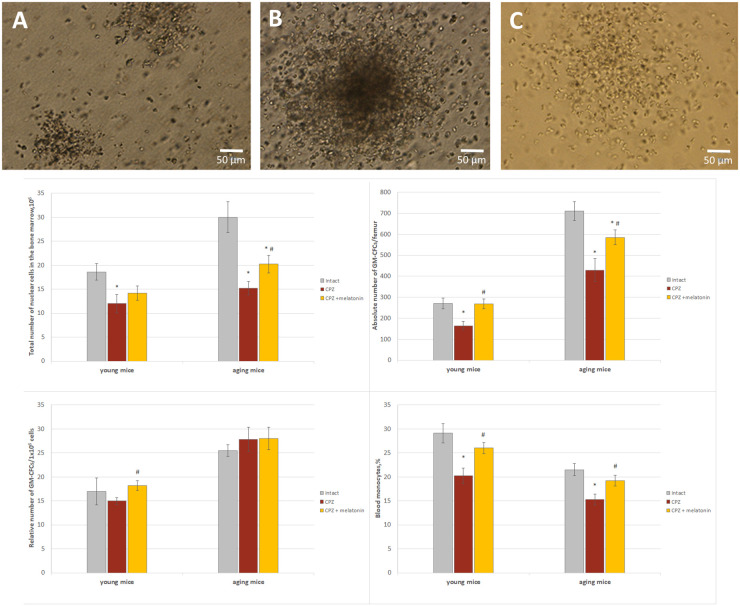
The number of GM-CFCs in bone marrow and the blood level of monocytes in young and aging mice of experimental groups. For GM-CFCs (*n* = 5) for monocytes (*n* = 10) from each group of mice. Types of GM-CFCs colonies: **(A)** granulocytic; **(B)** mixed; **(C)** macrophage. Phase contrast. Scale bar, 50 μm. Represented data are in 3 weeks of CPZ diet. Data are *M* ± SEM. *p < 0.05 compared to intact group; ^#^*p* < 0.05 compared to CPZ (*t*-test).

We found that, after 7 days of cuprizone administration, the amount of GM-CFCs in the bone marrow and monocytes in the blood of young (*n* = 4) and aging (*n* = 4) mice was not different from the intact animals (data not shown). After 3 weeks of cuprizone diet, the total number of nuclear cells and the absolute number of GM-CFCs were reduced in the bone marrow of young and aging mice compared to intact animals (Figure 9). Relative and absolute number of GM-CFCs in bone marrow in young mice after melatonin injections were higher than in cuprizone-treated mice (Figure 9). After administration of melatonin, the total number of nuclear cells and the absolute number of GM-CFCs in aging mice increased compared to cuprizone-treated mice, but remained at lower levels compared to intact animals.

Thus, under the influence of melatonin, the absolute number of GM-CFCs in the bone marrow of cuprizone-treated young mice changed due to an increase their colony-forming ability, but in cuprizone-treated aging mice, the absolute number of GM-CFCs increased due to elevation the total number of nuclear cells.

In addition, to confirm the positive effect only of melatonin on bone marrow function mice, the number of bone marrow GM-CFCs was measured in young and aging mice with regular diet after melatonin injections. Total number of nuclear cells in bone marrow of young mice treated with solvent (*n* = 8) or melatonin (*n* = 8) was 13.2 ± 1.7.10^6^ and 13.4 ± 1.5.10^6^, respectively; relative number of GM-CFCs was 21.0 ± 1.1/10^6^ and 25.0 ± 1.2/10^6^, respectively, (*p* < 0.05); absolute number of GM-CFCs was 275.1 ± 16.0/femur and 332.5 ± 23.0/femur, respectively, (*p* < 0.05). Total number of nuclear cells in bone marrow of aging mice treated with solvent (*n* = 8) or melatonin (*n* = 8) was 20.4 ± 1.2.10^6^ and 25.4 ± 1.8.10^6^, respectively (*p* < 0.05); relative number of GM-CFCs was 28.5 ± 2.1/10^6^ and 31.4 ± 3.2/10^6^, respectively; absolute number GM-CFCs was 581.4 ± 25.2/femur and 796.5 ± 28.5/femur, respectively (*p* < 0.05). Thus, melatonin alone increases the colony-forming ability of GM-CFCs in young mice and total number nuclear cells of bone marrow in aging mice with regular diet.

The positive effect of melatonin on the reduced number of GM-CFCs in the bone marrow in cuprizone-treated mice of different ages can be explained by the effects of melatonin itself on bone marrow cells in mice with a regular diet.

After 3 weeks of the cuprizone diet, the number of blood monocytes was reduced in mice of both ages compared to intact animals (Figure 9). After melatonin injections, the number of blood monocytes in both age mice increased compared to cuprizone-treated mice and did not differ from that of intact animals (Figure 9). Thus, melatonin positively affects on the number of blood monocytes in young and aging mice treated with cuprizone.

#### Level of thymulin in the blood

**FIGURE 10:**
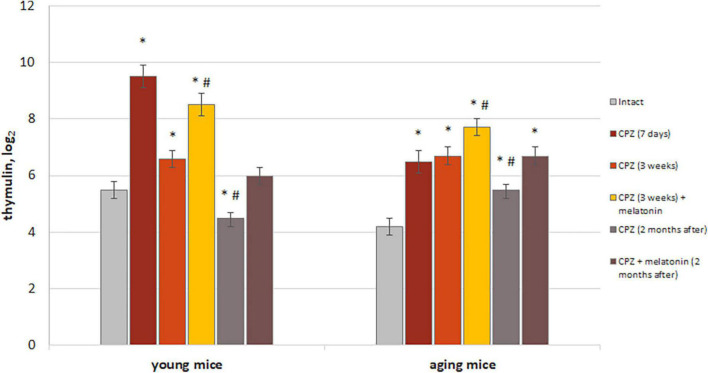
The effect of cuprizone (CPZ) and CPZ + melatonin on blood thymulin level in young and aging mice of experimental groups (*n* = 5 in each group). Represented data are in 7 days, 3 weeks of CPZ diet and 2 months after. Data are *M* ± SEM. *p < 0.05 compared to intact group; ^#^*p* < 0.05 compared to 3 weeks of CPZ (*t*-test).

Differentiation of T cell occurs in the thymus throughout life and is regulated by thymulin, one of the highly active thymic hormones (Bach et al., 1978; Csaba, 2016). We studied the dynamics of changes in level of thymulin in the blood of young and aging cuprizone-treated mice and after melatonin injection (Figure 10).

We found increased levels of thymulin in the blood of both young and aging mice after 7 days with cuprizone diet compared to intact animals. These increases were more pronounced in young mice than in aging mice treated with cuprizone. After 3 weeks of cuprizone diet in young mice level of thymulin was reduced compared to value after 7 days of application and also was lower than in intact ones in 2 months after completion of the cuprizone diet. Aging mice had higher blood thymulin level than intact mice after 3 weeks of the cuprizone diet and in 2 months after its completion. Thus, the dynamics of changes in levels of thymulin in aging mice is different from that in young mice during and after cuprizone administration.

Melatonin injections resulted in a significant increase levels of thymulin in blood in mice of both ages compared to mice treated with only cuprizone for 3 weeks. Two months after completion of administration of cuprizone and melatonin, levels of thymulin in blood of young mice decreased to those in intact animals but remained high in aging animals. Thereby, melatonin injection activates thymic function in cuprizone-treated mice of different ages, but only in young mice, thymic function is restored in 2 months after completion of cuprizone diet.

In addition, to confirm the positive effect only of melatonin on thymus function, level of thymulin in blood was measured in mice of both ages with a regular diet after melatonin injections. Levels of thymulin in blood in young mice treated with solvent (*n* = 8) or melatonin (*n* = 8) were 4.4 ± 0.4 and 6.2 ± 0.6, respectively (*p* < 0.05). Levels of thymulin in blood of aging mice treated with solvent (*n* = 8) or melatonin (*n* = 8) were 3.5 ± 0.3 and 6.0 ± 0.2, respectively (*p* < 0.05).

Thus, exogenous melatonin activated of thymus endocrine function in both young and aging mice with regular diet. The positive effect of melatonin on level thymulin in blood in cuprizone-treated mice of different ages can be explained by the effects of melatonin itself on thymus function in mice with regular diet.

## Discussion

### Reaction of different types of the brain cells on neurotoxin cuprizone in the young and aging mice

#### Brain cells of young mice

According to the authors, changes in the function of oligodendrocytes, their apoptosis and consequently the development of demyelination of nerve fibers occur in the brain of young mice during the early stages of cuprizone action (Praet et al., 2014; Vega-Riquer et al., 2019; Zirngibl et al., 2022). Demyelination pathology also refers to degenerative changes in brain neurons (Dutta and Trapp, 2011). Our previous study showed that after administration of cuprizone (within 3–4 weeks) in young 129/Sv mice, not only demyelination occurred, but also changes in the neuronal structure of the cerebral cortex, cerebellum, and hypocampus (increase the number of neurons with destructive and moderate changes) (Labunets et al., 2017a, 2018a,b, 2021).

The authors’ studies have shown that in young animals, changes in the structure and functioning of nerve cells under the influence of cuprizone can be associated with the development of oxidative stress and an increased production of pro-inflammatory cytokines by neuroinflammatory cells (Gudi et al., 2014; Vega-Riquer et al., 2019; Zhan et al., 2020). Therefore, we found it necessary to study and compare the dynamics of changes in the neurons structure and the number of microglial/macrophage cells, T cells, astrocytes in the brain both young and aging cuprizone-treated mice. Previously we established that after 7 days of cuprizone taking, the number of neurons with moderate structural changes significantly increased in the cortex and cerebellum of young mice (Labunets et al., 2018a,^2021^). As it turned out such changes in neurons coincide with an increased number and activity of phagocytic macrophages in the brain. The activating effect of neurotoxin on macrophages of young mice persisted even after 3 weeks of cuprizone diet, when the number of neurons with destructive changes in the brain sharply increased.

According to Praet et al. (2014), not only an increase in the number of activated microglial/macrophage cells but also in their interaction with T lymphocytes are important for the development of nerve cell damage in the brain. It has been shown that in the CNS both CD4+ and CD8+ T cells are involved in the development of demyelination as a result of production by these cells of pro-inflammatory cytokines IFN-γ and TNF-α (Sonobe et al., 2007; Almuslehi et al., 2020). Besides, CD4+ T cells also produce IL-17, an important factor in the development of experimental demyelination, including cuprizone-induced (Sonobe et al., 2007; Kang et al., 2012). In our experiments, we have shown that the number of CD3+, CD3+CD4+ and CD3+CD8+ T cells significantly increased in the brain of young mice after 3 weeks of taking cuprizone. We do not rule out that such changes in the number of CD3+ T cells are the result of an increase in the number of activated macrophages during the early period of cuprizone treatment (in 7 days). Pro-inflammatory cytokines (TNF-α, IFN-γ, and IL-1β) produced by activated microglia/macrophages increase the expression of adhesion molecules in microvascular endothelial cells, leading to passage of T lymphocytes through the BBB (Gonzalez and Pacheco, 2014).

An important issue is the state of the BBB in mice with the cuprizone model of demyelination and the possibility of different origins cells penetrating through it into the brain. A number of researchers point to intact BBB in mice with the cuprizone diet, thereby excluding this possibility (Skripuletz et al., 2011; Tejedor et al., 2017). However, there are literature data supporting this possibility. The authors have shown that already at early stages of the development of multiple sclerosis, accompanied by neuroinflammation, the permeability of the BBB increases (Kamphuis et al., 2015). The increase of the permeability of the BBB was found already after three days of cuprizone diet (Shelestak et al., 2020). In addition, immune cells with a peripheral phenotype were found in the brains of the cuprizone-fed mice (McMahon et al., 2002).

Our experiments revealed a simultaneous significant increase in the number of CD3+CD4+ T lymphocytes as well as the development of reactive gliosis in young mice after 3 weeks of taking cuprizone. A study by Kang et al. (2012) showed that IL-17 produced by brain CD4+ T cells affects astrocytes and stimulates the synthesis of proinflammatory cytokines in astrocytes after 3–4 weeks of cuprizone diet. Other studies also point to the interaction of lymphocytes and astrocytes during the development of demyelination and neurodegeneration (Absinta et al., 2021).

#### Brain cells of aging mice

We found changes not only in the neurons, but also in neuroinflammatory cells in the brain of aging cuprizone-treated mice compared to young mice. Namely: a decrease in the percentage of neurons with destructive changes and, conversely, an increase in the proportion of neurons with moderate structural changes; delayed activation of macrophages and slightly increase in the number of CD3+CD8+ T lymphocytes.

The revealed features of the response of brain cells of aging mice to the damaging effects of cuprizone can be associated with age-related changes in both the cells themselves and in their interaction. It is known, that neurogenesis and the number of unchanged neurons in the mouse brain decrease with age, while increase the number of T lymphocytes; the proliferation and activity of microglial cells; the expression of ICAM-1 and chemokines (MCP-1 and MIP1α) produced by astrocytes and the permeability of the BBB (Gemechu and Bentivoglio, 2012; Hamilton et al., 2013; Moraga et al., 2015; Labunets et al., 2017a).

We also observed decrease of the brain unchanged neurons in the intact aging mice versus young animals, increase of number of CD3+, CD3+CD8+ T cells, change in the balance of CD3+CD4+/CD3+CD8+ cells and a decrease in the number of activated macrophages (CD3+CD11b+). In addition, our experiments showed a delayed activation of macrophages in the brain of aging cuprizone-treated mice. It is possible that such response of macrophages in aging animals may be one of the reasons for a less pronounced increase in the number of CD3+ cells in mice after 3 weeks of cuprizone diet and, as a result, less significant disturbances in the structure of brain neurons compared to young animals with the same diet. In our studies we found that after cuprisone taking the number of neurons with destructive changes in the brain of aging animals was half that of young animals. Experiments of Xu et al. (2010) on old animals with an inflammation model confirmed the importance of initial activation of microglia/macrophage cells for subsequent infiltration of the brain by T cells.

#### Behavioral reactions

In our experiment, changes in the structure of neurons in mice with a cuprizone model of demyelination are accompanied with disturbance of behavioral responses. Disorders of motor, emotional, exploratory activities and muscle tone develop in young and aging cuprizone-treated mice. Manifestations of changes in behavior in such mice may be associated with the physiological characteristics of the CNS zones studied by us (cortex and hippocampus). The cerebral cortex together with cerebellum and the motor neurons of the spinal cord provide the nervous regulation of movement. In addition, the cerebral cortex together with the structures of the limbic system is an important component in the formation of emotional activity.

### Reaction of different types of brain cells of young and aging mice with a cuprizone model of demyelination to melatonin administration

#### Neurons

We have found that exogenous melatonin had a positive effect on the structure of neurons in the cerebral cortex of cuprizone-treated mice of both age groups. For example, in the cerebral cortex of mice the proportion of neurons with destructive changes significantly decreased and the proportion of unchanged neurons increased. Similar changes in the structure of neurons under the influence of melatonin were previously found by us in the cerebellum and hippocampus of mice with a cuprizone diet (Labunets et al., 2018a,^2021^).

The simultaneous decrease in the proportion of Nestin+ cells in the brain of such mice may be associated with their increased differentiation in the neurogenic direction. It is known ability of melatonin to penetrate the BBB, acting the viability, proliferation and differentiation of neural stem cells in neurogenic areas/niches of the brain and changes the synthesis of neurotrophic factors (BDNF and NGF) (Sarlak et al., 2013; Li et al., 2016; Kim et al., 2019).

The increase in the proportion of neurons with moderate changes in the cerebral cortex of cuprizone-treated mice under the influence of melatonin likely reflects the activation of regenerative processes in neurons that develop against on the background of neurotoxin intake. The authors Ng and Lee (2019) have shown that both the increase surface area of the nucleus and its swelling in neurons with moderate damage is associated with a compensatory reaction. These changes are necessary for the synthesis of group of proteins involved in the restoration of damaged brain structures. There are datà on the effect of melatonin on protein synthesis in cells (Liu et al., 2019).

In addition, the remyelinating effect of exogenous melatonin in the brain of mice with a cuprizone model of demyelination showed (Ghareghani et al., 2019; Kim et al., 2019; Vega-Riquer et al., 2019). Remyelinating action of melatonin *in vitro* in cuprizone-demyelinated brain culture was associated with an increased number of Olig2-cells and also with a change in the content of myelin basic protein (Rodnichenko and Labunets, 2018; Kim et al., 2019).

In our study, the less pronounced neuroprotective effect (structure of neurons and behavioral/functional reactions) of melatonin in aging mice with cuprizone model of demyelination can partly explain the age-related changes in the neurons themselves and their receptors to regulatory hormonal factors, in particular melatonin.

#### Neuroinflammatory cells

The protective effect of melatonin on the brain neurons and oligodendrocytes in demyelinating pathology is associated with its anti-inflammatory, anti-apoptotic and antioxidant effects (Vakilzadeh et al., 2016; Babaee et al., 2021; Murioz-Jurado et al., 2022).

The anti-inflammatory effect of melatonin is manifested in the inhibition of activation of NF-κB in cells, resulting to a decrease: (a) the synthesis of inflammatory mediators; (b) the level of adhesive molecules ICAM-1 and VCAM-1; (c) the production of pro-inflammatory cytokines (TNF-α, IL-1β, IFN-γ, IL-17, and IL-22) by T lymphocytes, macrophages and astrocytes and to an increase the production of anti-inflammatory cytokines (IL-10 and IL-4) (Escribano et al., 2014; Alvarez-Sanchez et al., 2015, 2017; Farez et al., 2015; Xu et al., 2018; Babaee et al., 2021; Murioz-Jurado et al., 2022). Under melatonin influence activity of T-helpers, T-effectors, T-regulatory cells changes and the differentiation of Th17 inhibits in animals with an experimental model of multiple sclerosis (Alvarez-Sanchez et al., 2015; Farez et al., 2015).

We have shown that after melatonin administration in young cuprizone-treated mice the number of T cells decreased. In addition, the number of activated macrophages and the number of activated astrocytes also decreased.

Less pronounced effects of melatonin on the number T cells and activation of macrophages in the brain of aging cuprizone-treated mice may be associated with age-related changes in the above cells and their receptors for melatonin (Gemechu and Bentivoglio, 2012; Hardeland, 2012; Hamilton et al., 2013; Moraga et al., 2015; Gimenez et al., 2022).

### Effects of cuprizone and melatonin on the oxidative stress, bone marrow, and thymus functions in young and aging mice

#### Oxidative stress of brain

It is known that oxidative stress products in demyelinating and neurodegenerative pathologies damage nerve cells (Chen et al., 2020; Murioz-Jurado et al., 2022). In cuprizone-induced demyelinating pathology, oxidative stress occurs in the brain, resulting in increased content of MDA and reactive oxygen species and decreased activity of antioxidant enzymes (Murioz-Jurado et al., 2022).

The properties of melatonin as a direct and indirect antioxidant in pathological processes of the CNS are described in reviews (Manchester et al., 2015; Chen et al., 2020). After melatonin injection the decrease in the content of oxidative stress biomarkers (including MDA) as well as an increase in the activity of antioxidant enzymes (superoxide dismutase, catalase, glutathione peroxidase, and glutathione reductase) were observed in the tissues of young animals with experimental models of multiple sclerosis (Abo Taleb and Alghamdi, 2020; Murioz-Jurado et al., 2022).

In our experiments we observed a decrease in the level of MDA in the brain tissue not only in young but also in aging mice with cuprizone demyelination model and injection of melatonin.

We have previously shown that reduced activity of glutathione peroxidase and glutathione reductase in the brain of young and aging cuprizone treated mice increased after melatonin injection (Labunets et al., 2018b; Labunets and Rodnichenko, 2020). As is known, the redox cycle of glutathione plays an important role in the antioxidant protection of the brain.

#### Bone marrow and brain macrophages

It has been shown that brain macrophages are a heterogeneous population consisting of resident and blood-derived cells (Jordan and Thomas, 1988; Li and Barres, 2018). In pathological conditions the number of macrophages in the brain may be increased by to peripherally derived macrophages (Cronk et al., 2018). Increase of the pool of macrophages with a peripheral immunophenotype (CD45+, Mac1+ cells) was found in the brain of young mice in cuprizone diet during 2–6 weeks (McMahon et al., 2002). Besides, in the pathology of the CNS it is possible that monocytes circulating in the blood enter the brain and differentiating into tissue macrophages (Zhao et al., 2020).

Since cells of the monocyte-macrophage series are of bone marrow origin, we estimated the number of GM-CFCs in the bone marrow of mice with a cuprizone diet as well as their change after melatonin administration. We found that in young and aging mice with a cuprizone diet, the amount of GM-CFCc in the bone marrow and the number of monocytes in the blood significantly decreased compared to intact animals. At the same time, the number of macrophages in the brain of such mice significantly increased. After melatonin injections the number of GM-CFCs in the bone marrow and circulating monocytes increased and the number of macrophages in the brain of both age groups was less than in the control group.

Under melatonin influence the number of not only GM-CFCs, but also more committed precursors of granulocytes, monocytes, and macrophages increased in bone marrow of adult mice (Maestroni, 1998; Currier et al., 2000). The stimulating effect of melatonin on hematopoiesis is realized directly through receptors in the cells of the monocyte/macrophage line as well as by increasing sensitivity of these cells to the effect of cytokines stimulating hematopoiesis (IL-3,4,6, granulocyte/macrophage-colony stimulating factor) (Currier et al., 2000; Garcia-Maurino et al., 2000).

Thus, it can be assumed that one of the possible ways of the influence of the neurotoxin cuprizone and hormone melatonin on the brain macrophages is associated with change in the functioning of the bone marrow.

#### Thymulin, T cells, and macrophages in the brain

As we have previously shown, thymulin affects *in vivo* and *in vitro* the number of GM-CFCs in the bone marrow and the functioning of macrophages together in young in aging animals (Labunets et al., 2017b). In addition, thymulin affects the differentiation of T lymphocytes in the thymus to CD4+ T cells and CD8+ T cells (Bach et al., 1978; Reggiani et al., 2014; Csaba, 2016; Savino et al., 2016). We suggested activation of the endocrine function of the thymus in cuprizone-treated mice of different ages is one of the possible links in the changes number of macrophages and T lymphocytes in the brain.

We showed the increase the level thymulin in blood in both age group of mice with cuprizone diet. Age-related differences in the dynamics of changes in thymulin level during cuprizone diet and remyelination period (in 2 months) may be associated with age-related dysfunction of the thymus as well as decreased number and/or affinity of receptors for glucocorticoids in the gland cells (Savino and Dardenne, 2000; Pazizandeh et al., 2004). It is known that glucorticoids, the level of which increases under the influence of damaging factors, have a depressing effect on the hormonal function of the thymus (Savino and Dardenne, 2000; Urbach-Ross and Kusnecov, 2007; Labunets, 2015).

Under the influence of melatonin, the level of thymulin was even higher in mice of both age groups compared with animals alone with cuprizone diet. Increasing of thymulin level in such mice seems to be aimed at enhancing the anti-inflammatory action of melatonin in the animal’s brain. This assumption is based on literature data about thymulin ability to increase the synthesis of anti-inflammatory cytokines (IL-10) and reduce the synthesis of pro-inflammatory cytokines (IL-1β, IL-6, and TNF-α) in the brain of animals with an experimental model of neuroinflammation (Haddad and Hanball, 2013). We observed in aging animals with cuprizone diet and melatonin injection the prolonged activation of the endocrine function of the thymus and no changes in the number of T cells during remyelination period (in 2 months) compared to those in young animals.

## Conclusion

The damaging effect of cuprizone on the structure and function of brain neurons of young and aging mice also accompanied with changes in the number and activity macrophages, T cells and astrocytes.

Neuroprotective effects of melatonin in the mice with cuprizone model of multiple sclerosis are associated with anti-inflammatory (decrease in the number and activity of macrophages, T lymphocytes and astrocytes) and antioxidant actions (decrease in MDA content). The anti-inflammatory effect of melatonin in the brain of mice with cuprizone diet is largely mediated by improving thymus and bone marrow functions.

The obtained results may be useful in the development of new biotechnological approaches to prevention and treatment of demyelinating pathology, in particular, multiple sclerosis.

## Data availability statement

The original contributions presented in this study are included in the article/supplementary material, further inquiries can be directed to the corresponding author.

## Ethics statement

This animal study was reviewed and approved by the Ethics Committee of the Institute of Genetic and Regenerative Medicine and performed in accordance with the European Union Directive of 22 September 2010 (2010/63/EU) for the protection of animals used for scientific purposes and Article 26 of the Law of Ukraine “On the Protection of Animals from Cruelty” (No. 3447-IV, 2006).

## Author contributions

IL: research concept and conceiving the study. IL, TP, AR, and SS: data collection, analysis, and interpretation of data. IL and TP: manuscript preparation and improving final version of manuscript. All authors read and approved the final manuscript.

## Footnotes

1
https://www.bioz.com/result/rabbitantiglialfibrillaryacidicprotein
